# Impact of Efavirenz on Heme Protein Induction and Biomarkers: Insights From In Vitro Experiments and a Clinical Study

**DOI:** 10.1111/cts.70526

**Published:** 2026-03-13

**Authors:** Leila Potzel, Jonny Kinzi, Isabell Seibert, Markus Grube, Werner Siegmund, Henriette E. Meyer zu Schwabedissen

**Affiliations:** ^1^ Biopharmacy, Department of Pharmaceutical Sciences University of Basel Basel Switzerland; ^2^ Center of Drug Absorption and Transport, Institute for Pharmacology, University Medicine Greifswald Greifswald Germany

**Keywords:** ALAS1, coproporphyrin I, CYP2B6, cytochrome P450 induction, efavirenz, endogenous biomarker, heme biosynthesis, MDCKII‐OATP1B1 cells, OATP1B1, transporter inhibition

## Abstract

Coproporphyrin I (CPI) is increasingly recognized as a reliable endogenous biomarker for hepatic OATP1B transporters. However, confounding mechanisms such as increased heme biosynthesis driven by induction of CYP enzymes, which are hemoproteins, may affect CPI levels independently of OATP1B1 inhibition. The aim of this study was to investigate whether activation of nuclear receptors and subsequent CYP induction by efavirenz affects CPI levels in vitro and in vivo. We first evaluated the potential of efavirenz to interact with OATP1B1 in vitro. We then assessed efavirenz‐induced changes in vitro, analyzing the mRNA expression of *CYP2B6* and both mRNA and protein expression of ALAS1 (5‐aminolevulinate synthase 1), the rate‐limiting enzyme in heme biosynthesis. Additionally, we quantified CPI levels in the cell culture supernatant. Finally, we determined plasma CPI levels in healthy volunteers pre‐ and post‐induction with efavirenz. In transport studies, efavirenz did not alter OATP1B1‐mediated uptake of CPI, whereas rosuvastatin reduced their intracellular accumulation. Treatment with efavirenz significantly increased *CYP2B6* mRNA expression, confirming CYP induction in vitro, but had no effect on ALAS1 expression at either mRNA or protein level. Correspondingly, CPI levels in the cell supernatant remained unchanged after efavirenz treatment compared to solvent control. In vivo, plasma CPI levels were also unaffected by efavirenz. Our in vitro findings demonstrate that while efavirenz induces CYP expression, it does not impact ALAS1 expression or alter CPI levels, supported by our in vivo findings where efavirenz did not change plasma CPI, suggesting that CYP enzyme induction does not relevantly affect coproporphyrin levels.

**Study Highlights**

- What is the current knowledge on the topic?
  ○ Coproporphyrin I (CPI), a byproduct of heme biosynthesis, is an endogenous biomarker for OATP1B1‐mediated interactions. Changes in CPI levels are driven by transporter inhibition or by functional genetic variants affecting OATP1B1 activity.
- What question did this study address?
  ○ Does efavirenz‐driven heme protein induction influences CPI levels?
- What does this study add to our knowledge?
  ○ Efavirenz does not affect OATP1B1‐mediated transport of CPI but induces CYP2B6 and CYP3A4 in vitro. In vivo, long‐term efavirenz treatment leads to efficient CYP3A4 induction without altering CPI levels. Taken together, these findings demonstrate that CYP induction alone does not influence CPI levels.
- How might this change clinical pharmacology or translational science?
  ○ Our findings support the use of CPI as biomarker for OATP1B1 involving drug–drug interactions in clinical and early‐phase studies, excluding the impact of heme protein induction as confounding factor.

## 1 Introduction

The Organic Anion Transporting Polypeptides (OATP) 1B1 and 1B3 are highly expressed in the sinusoidal membrane of hepatocytes facilitating the hepatocellular entry of their substrates [1], whereby affecting drug efficacy and safety [2]. Particularly for OATP1B1, it has repeatedly been shown that altered transport function due to genetic polymorphisms results in increased systemic exposure of substrate drugs [3, 4, 5, 6]. However, not only function‐impairing genetic polymorphisms affect levels of OATP1B1 substrates, but also drug–drug interactions [7]. In addition to their role in drug disposition, OATP1B‐transporters also recognize endogenous molecules as substrates [8], among them are the coproporphyrins [9], coproporphyrin I (CPI) and coproporphyrin III (CPIII), of which CPI has evolved into an endogenous biomarker for drug–drug interactions involving OATP1B transporters [10]. From the first description in 2016, where CPI was shown to increase with exposure to cyclosporine or rifampicin in preclinical models [11], there is now extensive clinical evidence reinforcing the use of CPI measurements as a reliable marker for OATP1B1‐involving interactions and therefore the drug–drug interaction (DDI) risk assessment in early drug development [10, 12, 13].

Considering the endogenous origin of CPs as byproducts of heme biosynthesis, it raises the question whether changes in CPI levels can be caused by other mechanisms besides the inhibition of OATP1B transporters. One OATP1B‐inhibitor that has been applied in various studies providing clinical evidence for the increase of CPI exposure in its presence was the antituberculotic rifampicin. Single doses of this drug are used during drug development to determine drug–drug interactions involving OATP1B1 [14, 15, 16, 17]. Nevertheless, rifampicin not only interacts with OATP1B [18], but administered in multiple doses, it is also a potent activator of the Pregnane X receptor (PXR). Importantly, in 2023 Kinzi et al. [19] reported on CP serum levels in individuals induced by a 9‐day treatment with rifampicin (600 mg/day) observing high levels (two‐fold the baseline) even 14 h after the last rifampicin intake. This indicated that elevated CP levels after long‐term treatment with multiple doses of rifampicin cannot be solely attributed to OATP1B1 inhibition, as rifampicin's half‐life in the induced state is typically 1–2 h [20, 21], meaning its levels would be negligible at this time point after the last intake [22].

Rifampicin as a PXR‐inducer modulates the expression and activity of various genes involved in drug metabolism [23, 24] with pronounced inducing effects on various cytochrome P450 metabolizing (CYP) enzymes [25]. CYP enzymes are heme‐containing proteins, whose expression is modulated not only by PXR but also by various nuclear receptors, such as the Constitutive Androstane Receptor (CAR). In this study, we focused on efavirenz, a non‐nucleoside reverse transcriptase inhibitor that is also activating PXR and CAR [26], whereby efficiently inducing CYP enzyme expression upon long‐term treatment [27]. Alterations in CYP enzyme production due to nuclear receptor activation may influence the demand for heme and/or its biosynthesis, during which CPs are spontaneously formed as isomeric byproducts. Moreover, the rate‐limiting step of this synthesis is catalyzed by the 5‐aminolevulinate synthase 1 (ALAS1) [28], which is also transcriptionally regulated via nuclear receptors [28, 29].

Consequently, it may be hypothesized that higher production of heme proteins as expected under induced conditions may affect CPI levels. In this study, we aimed to address this hypothesis by testing whether a CYP‐inducing drug, which is no substrate or inhibitor of OATP1B, would also affect CPI levels. To investigate this further, we chose efavirenz, a known inducer of PXR and CAR [26] that regulates the expression of CYP enzymes (detailed schematic overview Figure S1) [27, 30].

We initially tested efavirenz for its interaction with OATP1B1 in vitro. We then performed induction experiments in a hepatic cell line to determine whether efavirenz induces mRNA expression of *CYP2B6* and the rate‐limiting enzyme ALAS1 on mRNA and protein levels. Furthermore, we used residual plasma samples from a clinical study [27, 31], to compare levels of the biomarker CPI pre‐ and post‐induction with efavirenz.

## 2 Materials and Methods

### 2.1 Transport Experiments

Cellular transport experiments were performed as described previously [32]. Briefly, MDCKII or MDCKII‐OATP1B1 cells were cultured in DMEM (Sigma‐Aldrich, Buchs, Switzerland) supplemented with 10% Fetal calf serum (FCS, BioConcept, Allschwil, Switzerland) and 2 mM L‐Glutamine (BioConcept). 500 μg/mL Geneticin (Carl Roth, Arlesheim, Switzerland) was added to the medium of MDCKII‐OATP1B1 cells. For transport experiments, cells were seeded at a density of 75,000 cells/well in 24‐well plates. The day after seeding, cells were stimulated with 2 mM sodium butyrate for 24 h. Then transport experiments were initiated by washing the cells with phosphate‐buffered saline (PBS) followed by a 10‐min equilibration in Hanks‐buffer (Sigma‐Aldrich) supplemented with 10 mM HEPES (HBSSH, BioConcept). After equilibration, the supernatant was exchanged to HBSSH supplemented with either 1 μM CPI or 1 μM CPIII, and efavirenz at a concentration ranging from 0.01 μM to 10 μM. As control, 50 μM rosuvastatin was added instead of efavirenz. After 10 min exposure at 37°C in the dark, the cells were thoroughly washed with ice‐cold PBS and subsequently lysed in 1 mM HEPES‐1% Triton X‐100, pH 6.5 (lysis buffer) for 30 min at 37°C. A 10‐μL aliquot of lysate was applied for protein quantification using the Pierce BCA Protein Assay Kit (ThermoFisherScientific, Reinach, Switzerland). In the remaining lysate, CPI or CPIII were quantified after centrifugation at 540× *g* and 4°C for 10 min measuring the absorbance using the TECAN M200Pro (λex 395 nm and λem 620 nm) in 100‐μL sample. CPI or CPIII concentrations were calculated by linear regression applying a calibration curve ranging from 0.333 to 1000 nM in lysis buffer.

### 2.2 Treatment of HepaRG Cells for Expression Analyses

HepaRG cells were cultured as progenitor cells for 14 days in William's medium E (ThermoFisherScientific) containing 10% FCS, 2 mM L‐Glutamine, 5 μg/mL human insulin (Sigma‐Aldrich), 50 μM hydrocortisone hemisuccinate (Sigma‐Aldrich), and 100 units/mL penicillin/100 μg/mL streptomycin (BioConcept). For differentiation, 2% DMSO was added to the medium for 14 days. Differentiated HepaRG cells were treated with 10 μM efavirenz, 1 μM CITCO, or solvent control in William's medium E with 2% FCS for 72 h, with the treatment medium refreshed daily. Finally, cells were harvested for subsequent analysis of mRNA or protein expression.

For mRNA expression analysis, cells were lysed in TRI Reagent (Sigma‐Aldrich) followed by mRNA extraction according to the manufacturer's instructions. Content and quality of extracted mRNA was determined using the NanoDrop One instrument (ThermoFisherScientific) followed by reverse transcription with 1 μg of RNA and the High‐Capacity cDNA Reverse Transcription Kit (ThermoFisherScientific). Levels of *CYP2B6*, *ALAS1*, and *18S* expression were determined by real‐time PCR using the SYBR‐Green RT‐PCR‐Kit (ThermoFisherScientific), specific primers (Table S1), and the QuantStudio 5 (equipped with the Design & Analysis Software v.1.5.1; ThermoFisherScientific). Gene expression was evaluated using the 2^−ΔΔCT^ method [33].

For protein expression analysis, cells were harvested in 500 μL of 5 mM TRIS–HCl (pH 7.4) supplemented with protease‐inhibitor cocktail (Sigma‐Aldrich). The tubes underwent 5 freeze–thaw cycles and the lysate was then centrifuged at 700× *g* for 5 min at 4°C to remove cell debris. Subsequently, the supernatant, which contained the total protein, was used for further analysis. After measurement of the protein content, 10 μg of each sample was supplemented with 4× Laemmli and incubated at 37°C for 1 h. Proteins were then separated using a SDS 10%‐PAGE and the Mini‐PROTEAN Tetra cell system (Bio‐Rad Laboratories, Cressier, Switzerland) and were afterwards transferred onto a nitrocellulose membrane (Cytiva, Opfikon, Switzerland) using the Blot L1 system (GenScript, Piscataway, USA). Following Ponceau S staining to control transfer and separation, a 1‐h incubation at room temperature with 5% bovine serum albumin (BSA, Carl Roth) in TBS‐T (Tris‐buffered saline (TBS; 138.9 mM NaCl, 2.6 mM KCl, 25 mM Tris) containing 0.1% Tween20) was performed and the membrane was then exposed to the anti‐ALAS1 antibody, diluted 1:1000 in TBST‐5% BSA (ThermoFisherScientific; PA141152), overnight at 4°C. The next day, the membrane was washed several times with TBS‐T followed by incubation with the secondary antibody at room temperature for 1 h (goat anti‐rabbit 1:2000 in TBS‐T, Bio‐Rad Laboratories). The secondary antibody was visualized using the ChemiDocTM XRS imaging system equipped with the Image Laboratory software (Version 6.0) and Clarity Western ECL Substrate solution (Bio‐Rad Laboratories) according to manufacturer's instructions. Band intensity was densitometrically analyzed.

### 2.3 Clinical Study Samples

The herein reported clinical data were derived from 14 residual samples collected from healthy male volunteers (age 20–36 years) during a clinical study originally designed to evaluate the pharmacokinetic interaction between efavirenz (400 mg, Sustiva capsules; Bristol‐Myers Squibb, Munich, Germany) and ezetimibe (10 mg, Ezetrol tablets; MSD Sharp & Dohme, Haar, Germany). All participants gave written informed consent to the study and for further use of their biological material in the study reported herein. The study protocol as detailed before [27, 31] was approved on the 17th of December 2008 by the local ethics committee (ethics committee of the University medicine Greifswald, EudraCT 2009‐011050‐17).

The original study consisted of 4 periods. For the herein reported study, we selected samples from the study periods B and D. In period B, the participants were treated with ezetimibe only for 10 days (study days 6–15). In study period D (study days 21–36) participants were treated with ezetimibe and efavirenz simultaneously. Venous blood samples collected on study day 15 (steady state of ezetimibe) before daily ezetimibe treatment (*t* = 0 h) and on study day 30 (steady state of ezetimibe and efavirenz) before daily administration of both drugs (*t* = 0 h) were used for the measurement of CPI levels. Participants initiated efavirenz treatment on study day 21 and received efavirenz for 9 days to reach steady state by day 30. In addition, total bilirubin and 4β‐hydroxycholesterol plasma levels, available in the clinical report forms, were re‐evaluated in the context of our research question. Further information on the sample selection can be found in Figure S2.

### 2.4 Quantification of CPs in Clinical Samples and in Cell Culture Medium

CPI and CPIII were extracted from plasma samples and supernatants of differentiated HepaRG cells and measured using a method previously validated in our laboratory [19]. Briefly, CPs were quantified over a calibration range of 0.25–20 ng/mL using a UPLC equipped with an ACQUITY UPLC BEH C18 column (1.7 μm, 2.1 × 100 mm; Waters, Baden, Switzerland) for chromatographic separation, coupled with a G6460A triple quadrupole mass spectrometer (Agilent Technologies, Basel, Switzerland). Multiple reaction monitoring positive mode was used to measure the transitions for quantification of CPs (655.3 → 596.3, 655.3 → 537.3) and the internal standard [^15^N_4_]‐CPIII (Toronto Research Chemicals, North York, ON, Canada) (659.6 → 467.2). For sample preparation, 200 μL plasma or cell supernatant were mixed with 200 μL of 1.25% ammonium hydroxide (NH_4_OH) containing [^15^N_4_]‐CPIII as internal standard. For solid‐phase extraction, the Oasis MAX 96‐well μElution plate (30 μm; Waters) was used together with a positive pressure manifold with three to six bar. Prior to sample loading, the plates were conditioned with 500 μL methanol followed by equilibration with two times 500 μL of Milli‐Q water. After that, diluted samples were loaded onto the SPE columns. Columns were then washed with 500 μL of 1.25% ammonium hydroxide (NH_4_OH) followed by 500 μL of methanol. Elution was performed with two times 500 μL acetonitrile/methanol/formic acid (47.5:47.4:5, *v*:*v*:*v*). After collection, the eluate was dried at 50°C for 60 min under constant nitrogen flow (Evaporex EVX‐96; Apricot Designs, Melbourn, UK), residues were then reconstituted in 100 μL acetonitrile/methanol/formic acid (47.5:47.4:5), prior to the injection of 20 μL for each sample.

### 2.5 Data Analysis

Data analysis was conducted using GraphPad Prism (Version 10.2.0 for Windows, San Diego CA, USA). Transport experiments were statistically analyzed using an unpaired *t*‐test, assuming equal standard deviations, or an Ordinary one‐way ANOVA test. Statistical differences in mRNA expression were assessed using a Kruskal–Wallis test. A Brown–Forsythe ANOVA test was applied to compare CPI and CPIII levels in the supernatant of cells before treatment with efavirenz and after. Statistical comparison of CPI concentrations prior to efavirenz induction and following in vivo induction was performed using an unpaired *t*‐test with Welch's correction. A paired analysis was not feasible owing to incomplete paired sample sets due to insufficient sample volume and/or sample degradation. The corresponding statistical test and the *p*‐value are stated within the context of the results. If applicable, a Dunn's or Dunnett's multiple comparisons test was applied following a significant Kruskal–Wallis or ANOVA test, respectively.

## 3 Results

### 3.1 Interaction of Efavirenz with the OATP1B1‐Mediated CPI and CPIII Uptake

**FIGURE 1:**
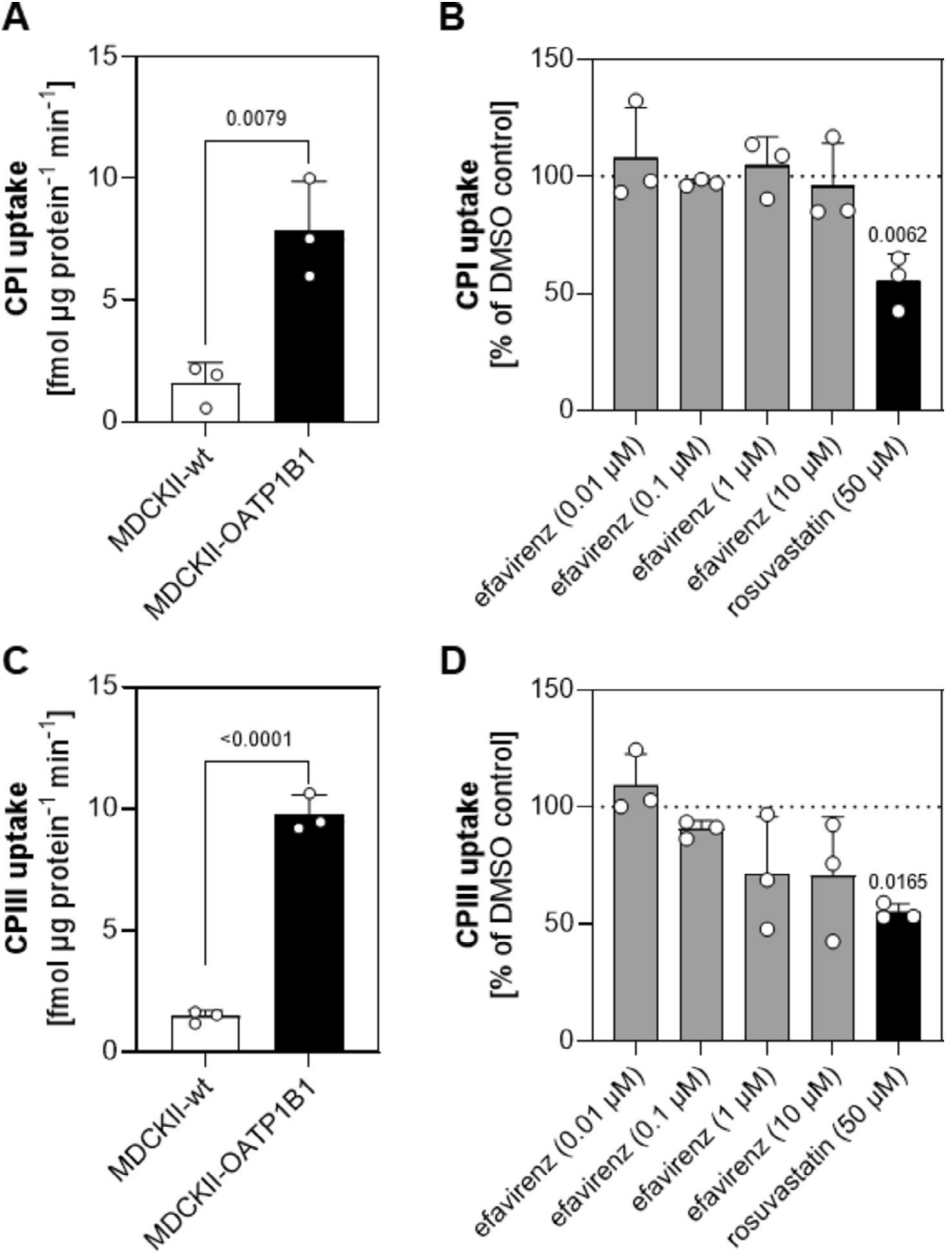
Interaction of efavirenz with the OATP1B1‐mediated uptake of CPs. MDCKII or MDCKII‐OATP1B1 cells were exposed for 10 min to coproporphyrin I (CPI, A) or coproporphyrin III (CPIII, C) to determine the transport rate. Data are reported as mean ± SD [fmol/μg protein/min] of *n* = 3 independent experiments, each performed in technical triplicates. For statistical analysis, an unpaired *t*‐test was applied; CPI: *p* = 0.0079, CPIII: *p* < 0.0001. The impact of efavirenz on the OATP1B1‐mediated uptake of CPI (B) or CPIII (D) was tested in MDCKII‐OATP1B1 cells. The OATP1B1‐substrate rosuvastatin was applied as control. Data are reported as the mean % of solvent control ± SD of *n* = 3 independent experiments, each performed in technical triplicates. For statistical analysis, an Ordinary one‐way ANOVA with Dunnett's multiple comparisons test was applied.

Initially, we assessed the influence of efavirenz on the OATP1B1‐mediated uptake of CPs. Compared to MDCKII control cells, we observed a five‐fold higher uptake rate for CPI in MDCKII‐OATP1B1 cells (mean CPI‐uptake rate ± SD [fmol/μg protein/min]; MDCKII vs. MDCKII‐OATP1B1; 1.555 ± 0.875 vs. 7.846 ± 2.036; unpaired *t*‐test; *p* = 0.0079; Figure 1A). As shown in Figure 1B, the presence of rosuvastatin significantly reduced the OATP1B1‐mediated cellular accumulation of CPI (mean CPI‐uptake as % of DMSO control ± SD; rosuvastatin 50 μM; 55.31 ± 11.53 vs. DMSO; Ordinary one‐way ANOVA with Dunnett's multiple comparisons test; *p* = 0.0062), while no such effect was observed for efavirenz at concentrations ranging from 0.01 μM to 10 μM (mean CPI‐uptake as % of DMSO control ± SD; efavirenz 10 μM 95.86 ± 18.31 vs. DMSO, *p* = 0.9942; efavirenz 1 μM 104.5 ± 12.27 vs. DMSO, *p* = 0.9915; efavirenz 0.1 μM 97.43 ± 1.410 vs. DMSO, *p* = 0.9994; efavirenz 0.01 μM 108.0 ± 21.39 vs. DMSO, *p* = 0.9158). Similar results were obtained when testing interaction of efavirenz with the OATP1B1‐mediated uptake of CPIII (Figure 1D), where we observed a seven‐fold higher accumulation in MDCKII‐OATP1B1 cells compared to controls (mean CPIII‐uptake rate ± SD in fmol/μg protein/min; MDCKII vs. MDCKII‐OATP1B1; 1.466 ± 0.245 vs. 9.790 ± 0.7615; unpaired *t*‐test; *p* < 0.0001, Figure 1C). The observed increase in CP uptake in MDCKII‐OATP1B1 cells relative to MDCKII wild‐type cells is comparable to the enhanced cellular accumulation of the well‐known substrate estrone‐3‐sulfate (E1S) that is about seven‐fold, measured applying the same cellular system in our laboratory (data not shown).

### 3.2 Influence of Efavirenz Treatment on the mRNA and Protein Expression of ALAS1 and CP Content in the Cell Supernatant

**FIGURE 2:**
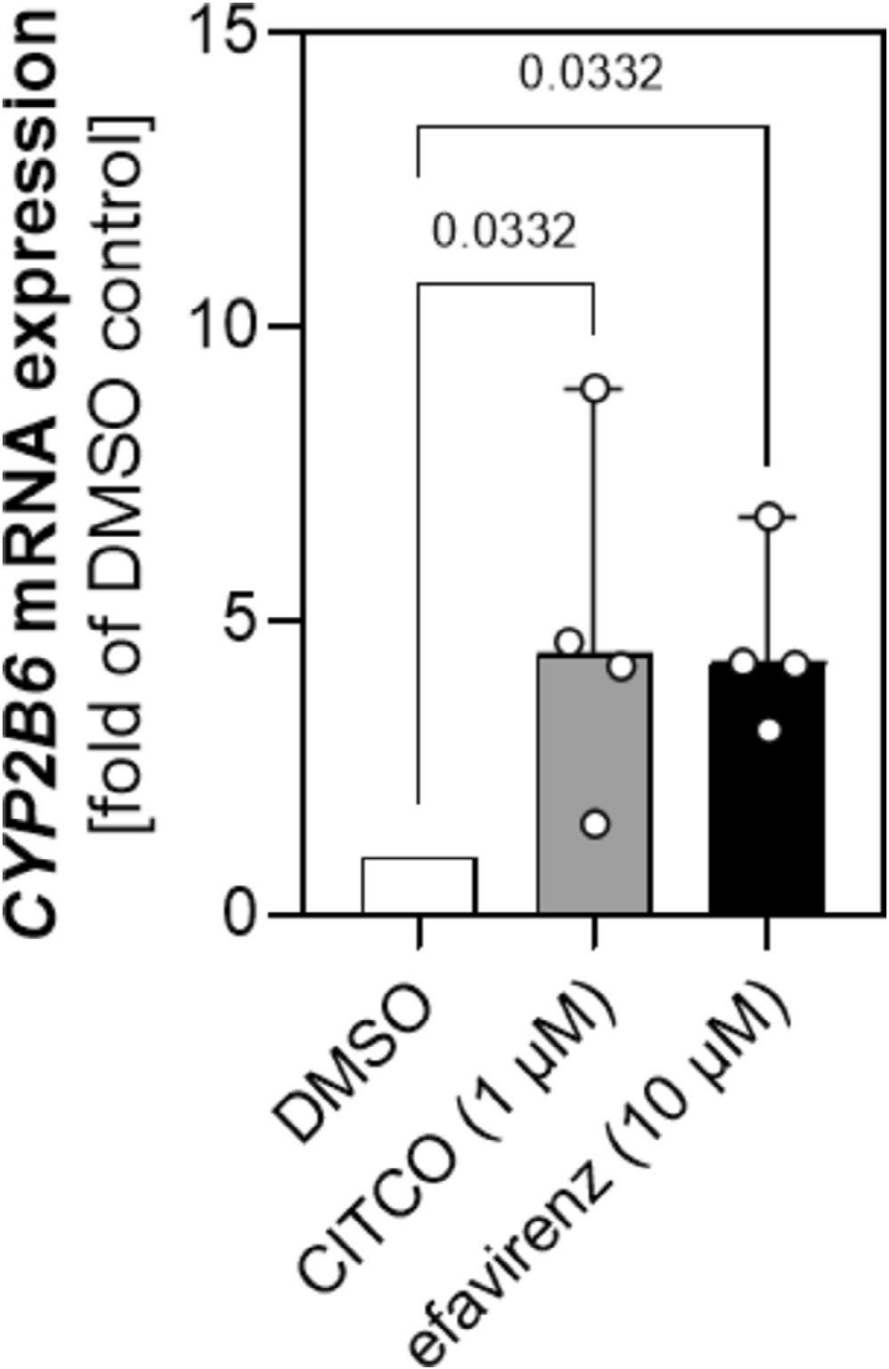
Influence of efavirenz treatment on the mRNA expression of *CYP2B6*. Differentiated HepaRG cells were exposed for 72 h to 10 μM efavirenz, 1 μM CITCO, and 0.1% DMSO (solvent control) to determine the influence on *CYP2B6* mRNA expression. Data are reported as the median with 95% CI as fold of DMSO of *n* = 4 independent experiments, each performed in technical duplicates. For statistical analysis, a Kruskal–Wallis with Dunn's multiple comparisons test was applied.

**FIGURE 3:**
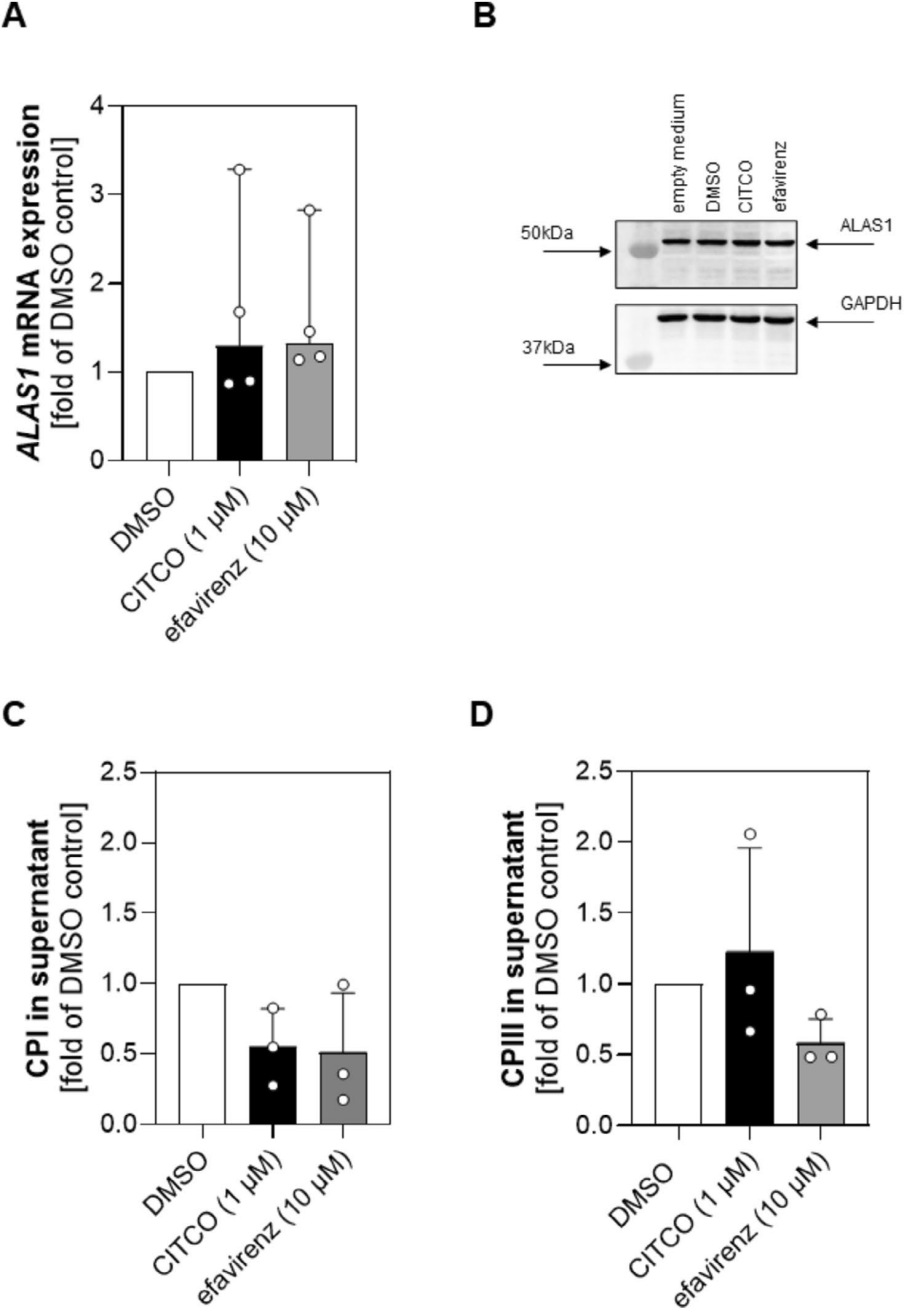
Influence of efavirenz treatment on the mRNA and protein expression of ALAS1 and CP content in the cell supernatant. Differentiated HepaRG cells were exposed for 72 h to 10 μM efavirenz, 1 μM CITCO and 0.1% DMSO (solvent control) to determine the influence on mRNA (A) and protein expression (B) of ALAS1 and on the CPI (C) and CPIII (D) content in the cell supernatant. Data for *ALAS1* mRNA expression (A) are reported as the median, with 95% CI as the fold of solvent control DMSO of *n* = 4 independent experiments, each performed in technical duplicates. For statistical analysis, a Kruskal–Wallis test was applied. Data (B–D) are reported as mean ± SD as the fold of solvent control DMSO of *n* = 3 independent experiments. For statistical analysis, a Brown–Forsythe ANOVA test was applied.

Next, we intended to determine whether treatment with efavirenz affects heme biosynthesis and therefore production of CPI in a hepatic immortalized cell line. Applying differentiated HepaRG cells, we first validated efficient induction by measuring the expression of the well‐known CAR‐target gene *CYP2B6*. After treatment with 10 μM efavirenz, analysis of mRNA expression revealed a significant increase in *CYP2B6* expression (Figure 2) (median [95% CI] fold of DMSO control; 4.266 [3.137 to 6.772], Kruskal–Wallis with Dunn's multiple comparisons test, *p* = 0.0332). Similar results (4.420 [1.533 to 8.956], Kruskal–Wallis with Dunn's multiple comparisons test, *p* = 0.0332) were obtained for CITCO which is commonly applied as control in in vitro studies. However, efavirenz treatment did not lead to a significant change in mRNA expression of the rate‐limiting enzyme *ALAS1* (median [95% CI] fold of DMSO control; 1.319 [1.145 to 2.827], Kruskal–Wallis test, *p* = 0.1794) (Figure 3A). The observation that CAR‐activation does not affect *ALAS1* expression is further supported by our findings on protein level as depicted in the Western blot in Figure 3B. Additionally, when performing the densitometrical analysis, no significant impact of efavirenz or CITCO on ALAS1 abundance was observed (mean fold of DMSO control ± SD; Ordinary one‐way ANOVA test; *p* = 0.2409; efavirenz: 1.354 ± 0.2781, CITCO: 1.294 ± 0.1492). Nevertheless, we also quantified the levels of CPs in the supernatant of the cultured cells. As shown in Figure 3C,D, there was no significant change of neither CPI (C) nor CPIII (D) in presence of efavirenz (mean CPI in nM ± SD: 0.08497 ± 0.06986; mean CPIII in nM ± SD: 0.9611 ± 0.5959) or CITCO (mean CPI in nM ± SD: 0.07864 ± 0.04387; mean CPIII in nM ± SD: 1.790 ± 0.9111) and DMSO (mean CPI in nM ± SD: 0.1599 ± 0.08804; mean CPIII in nM ± SD: 1.749 ± 1.326; Brown‐Forsythe ANOVA test, CPI *p* = 0.3615, CPIII *p* = 0.5578), respectively.

### 3.3 Influence of Multiple Dose Efavirenz Treatment on CP Plasma Levels

**FIGURE 4:**
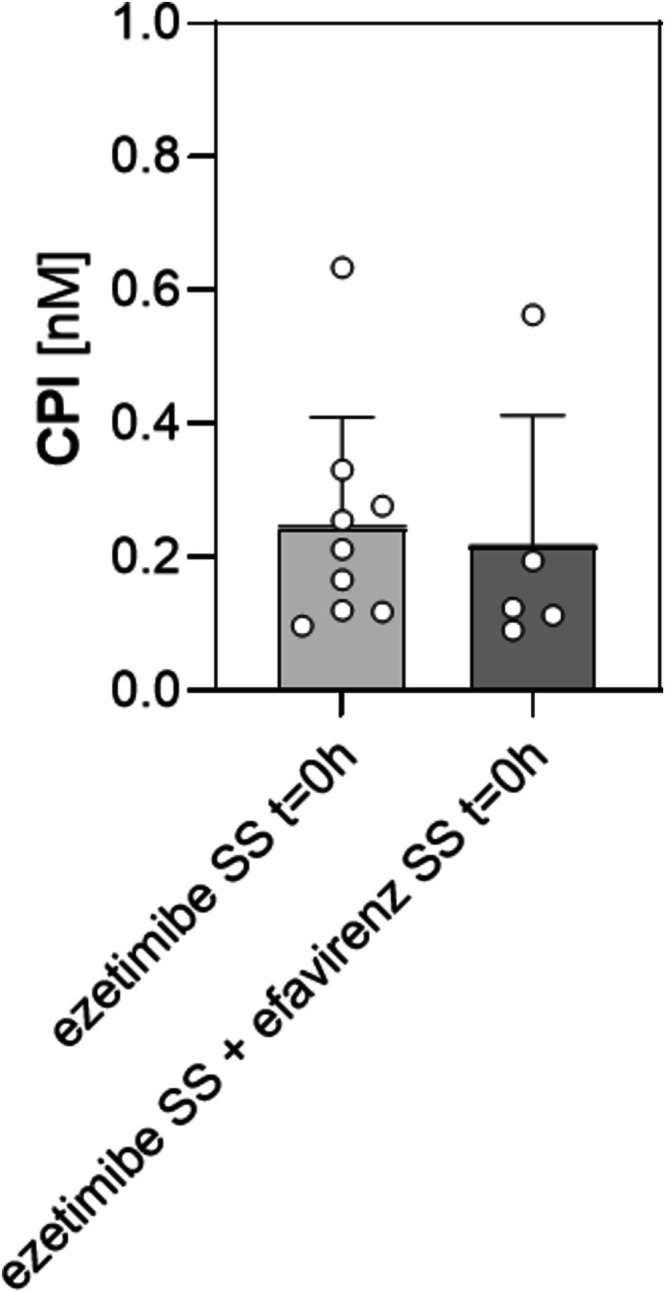
CPI plasma levels pre‐ and post‐efavirenz induction. CPI plasma levels were determined in volunteers before and after oral treatment with efavirenz (400 mg once daily) for 9 days. Reported are CPI levels in the steady state of ezetimibe (ezetimibe SS *t* = 0 h) and in the steady state of ezetimibe and efavirenz (ezetimibe SS + efavirenz SS *t* = 0 h) prior to ezetimibe administration. Blood samples were drawn before drug administration of the respective day. Data are presented as mean ± SD. An unpaired *t*‐test with Welch's correction was used to evaluate differences.

To determine whether induction of CYP enzymes would affect CPI levels in humans, we quantified CPI in residual samples of healthy volunteers treated with efavirenz (400 mg once daily) for 9 days. The original study tested the impact of efavirenz induction on steady state ezetimibe, accordingly we are not reporting on treatment naive values in our comparison [31]. Efficient induction of CYP3A4 can be verified, illustrated by the increase in 4β‐hydroxycholesterol levels compared to control [34]. For the individuals, where residual samples were available (day 15 *n* = 9; day 30 *n* = 5) and which are included in the herein reported CP measurement, the pre‐post induction assessment revealed mean 4β‐hydroxycholesterol levels of 5.356 ± 2.227 ng/mL at day 15 of the study. At this time point in the study, the individuals were in steady state for ezetimibe, but did not receive efavirenz. At day 30 of the study, at which the individuals were in steady state for ezetimibe and for efavirenz (fully induced state) the mean 4β‐hydroxycholesterol levels were 9.640 ± 4.497 ng/mL, revealing a significant increase by about 80% (unpaired *t*‐test, *p* = 0.0321, Figure S3) in this biomarker of CYP3A4‐activity. Total bilirubin levels were also previously determined [27]. The samples included in the present analysis showed a reduction by 30% (mean total bilirubin concentration ± SD [μM] in the steady state of ezetimibe vs. steady state of ezetimibe and efavirenz [fully induced state]; 9.067 ± 2.666 vs. 6.340 ± 2.038; unpaired *t‐*test, *p* = 0.0358, Figure S4). Applying the residual samples to a quantification of CPI, we observed no differences when comparing the levels pre‐ and post‐efavirenz induction (CPI pre‐induction vs. post‐induction: mean ± SD [nM]; 0.2442 ± 0.1664 vs. 0.2153 ± 0.1977, unpaired *t*‐test with Welch's correction, *p* = 0.7893, Figure 4).

## 4 Discussion

In this study, we selected efavirenz as a known inducer of cytochrome P450 enzymes to follow up on our previous finding where we reported increased levels of coproporphyrins (CPs) in individuals treated with multiple doses of rifampicin [19]. Specifically, in this previous study the subjects were administered rifampicin 600 mg once daily for 9 days, to ensure full CYP induction [35]. Measuring CPs, we observed higher levels compared to baseline at a time after the last dose of rifampicin [19, 32], where its levels are assumed to be negligible [22]. This observation led us to hypothesize that changes in CYP production and/or linked heme biosynthesis might influence CP levels, which may be of consequence for the application of CPI as a biomarker for drug–drug interactions involving OATP1B1 and CYP enzymes [13, 36]. In this context, it seems worth mentioning that rifampicin is a potent inducer of PXR, which has been reported previously to induce expression of ALAS1, the key enzyme of heme biosynthesis [28]. Moreover, CYP enzymes are heme‐containing proteins. Accordingly, their production is linked to the availability of heme and induction could alter heme biosynthesis. However, establishing whether this relationship influences CP concentrations, and thus its reliability as a biomarker, is challenging in rifampicin‐based studies, as rifampicin not only activates PXR but also inhibits OATP1B1. In contrast, the CAR activator efavirenz, for which no OATP1B1 interaction has been reported to date, may offer a clearer assessment. To rule out the possibility that efavirenz itself inhibits OATP1B1, we evaluated its effect on the in vitro transport of CPs. In our in vitro studies, we used efavirenz concentrations close to the reported peak plasma concentration in healthy individuals (9 μM [37]). Using OATP1B1‐overexpressing cells, we observed a significant reduction in cellular accumulation of both CPs when treated with 50 μM of the OATP1B1 substrate rosuvastatin [38]. While this concentration exceeds physiological plasma exposure (4.6 ± 2.1 ng/mL after 10 mg oral daily for 10 days [20]) and likely results in near‐saturating transporter conditions, it was selected to ensure robust detection of transporter‐mediated effects under controlled in vitro conditions. In contrast, efavirenz at all tested concentrations had no effect on both CP isomers (Figure 1), indicating that efavirenz does not inhibit OATP1B1. This conclusion is consistent with findings on the lack of inhibitory potency of efavirenz (1–1000 μM) when using bromosulfophthalein (BSP) as substrate of OATP1B1 [31].

Since excess of heme is toxic, heme biosynthesis is tightly regulated [39]. One mechanism known to alter heme content and biosynthesis in the liver is the modification of ALAS1 expression, which is the rate‐limiting enzyme of heme biosynthesis [28]. Moreover, it is known that *ALAS1* transcription is regulated by nuclear receptors including FXR, PXR and CAR [28, 29]. Even if there is evidence from a murine study [40] indicating PXR as the principal mediator of drug‐induced modulation of ALAS1, we sought to evaluate the effect of the CAR‐activator efavirenz on the regulation of this enzyme and its impact on CPI production. For our preceding in vitro analysis, we used the human liver cell line HepaRG, which endogenously expresses CAR and PXR and is well‐established as a model for hepatic induction studies [41]. Our results show that efavirenz does not influence *ALAS1* mRNA expression (Figure 3A), whereas it enhances *CYP2B6* mRNA expression (Figure 2). The magnitude of *CYP2B6* induction observed with efavirenz was similar to that achieved with the well‐established CAR agonist CITCO which was included as a benchmark for CAR‐mediated transcriptional activation and to verify the responsiveness of the experimental system. Notably, CYP2B6 is a well‐known CAR‐target gene and assessment of its mRNA levels is commonly applied to monitor the effects of this nuclear receptor on transcription [42, 43].

Moreover, we observed no impact of efavirenz on ALAS1 protein expression (Figure 3B), suggesting that the rate‐limiting enzyme in heme biosynthesis, where CPs are formed as spontaneous byproducts, remains unaffected by ligand‐mediated activation of CAR. This suggestion is further supported by our findings on CP levels in the supernatant of efavirenz‐treated cells, where neither CPI nor CPIII levels showed any significant changes compared to cells treated with the solvent control (Figure 3C,D). Besides, we quantified heme levels in the supernatant of cultured cells and found no difference after exposure to efavirenz (data not shown), suggesting that increased production of CYP2B6 alone does not affect heme content/heme biosynthesis. This assumption is supported by previous findings showing that CYP production itself does not influence ALAS1 in a heterologous expression system [44]. Irrespective of these observations, we proceeded with the measurement of CPI in plasma of healthy volunteers treated with multiple doses of efavirenz. In this regard, it seems worth mentioning that the majority (85%) of heme is produced in erythroid cells. Accordingly, it is expected that this is the main source of CPs in plasma [45]. In erythroid cells, the ALAS2 isoform is the key enzyme of heme biosynthesis, for which there is currently no information on whether it is inducible by PXR or CAR‐associated mechanisms. Although ALAS2 could contribute to circulating CPI, our study focused on hepatic heme biosynthesis involving the hepatic ALAS1 isoform. ALAS2 regulation was therefore beyond the scope of this study.

Quantifying CPI levels in residual plasma samples of healthy volunteers revealed no significant impact of efavirenz when comparing pre‐ and post‐induction (Figure 4). As not all participants could contribute paired measurements due to insufficient sample volume or degradation processes, an unpaired *t*‐test was selected for statistical analysis. Additionally, given the small sample size, we conducted a power analysis which revealed low statistical power. We acknowledge that our analysis is clearly underpowered, particularly if expecting changes in levels similar to those previously reported for the rifampicin‐mediated induction. However, when assessed in the same subset of samples, there was a nearly 2‐fold increase in 4β‐hydroxycholesterol plasma levels reaching statistical significance (Figure S3). This analysis was conducted using the respective entries from the clinical report forms of the original study [27] and is considered as a verification of in vivo induction of the heme protein CYP3A4 [34, 46, 47]. A major limitation of this study is the use of residual material, potentially leading to insufficient sample volume as well as sample degradation processes associated with long term storage. Consequently, some aliquots were excluded from the analysis, and paired measurements were not available for all individuals. The resulting dataset contained both paired and unpaired observations, precluding exclusive use of a repeated‐measures framework. Although samples were stored under standardized conditions, including temperature‐monitoring, they had been stored for an extended period and thawed several times for previous analysis. Data from Ediage et al. [48] show that CPs are highly light sensitive. Their sun test CPS+ in plasma revealed a CPI loss of 44.1% in transparent tubes after 5 min of exposure which translates to a similar degradation after an exposure of half an hour of direct sunlight (UV). To monitor for that, detection of UV‐degradation products could be considered. However, due to the complex chemical structures of CPs, their degradation products cannot be precisely predicted as degradation may occur at various sites within the porphyrin ring, potentially leading to a large number of degradation products, so that we are unable to control for that. A limitation of the current bioanalytical approach is the use of [^15^N_4_]‐CPIII as the internal standard for CPI. While employing a labeled CPI internal standard would be ideal, the structural similarity and comparable chromatographic and ionization characteristics of CPI and CPIII suggest that any resulting deviation from the reported results is likely minimal. In the same subset of samples, we observed a considerable reduction in total plasma bilirubin levels (Figure S4). Notably, we had no information on hemoglobin levels in our study cohort to directly conclude on the heme status. Nevertheless, since bilirubin is the end product of heme degradation [49], this finding supports the notion that efavirenz‐mediated induction, characterized by increased CYP expression, indeed affects heme homeostasis. Specifically, it may suggest that heme residence time in the system is prolonged, with more heme being used for heme protein synthesis rather than being catabolized and eliminated.

Taken together, our findings—specifically, the absence of ALAS1 upregulation in vitro and the lack of changes in plasma CPI levels at full induction as verified by significantly increased 4β‐hydroxycholesterol levels following efavirenz treatment—suggest that CAR‐mediated induction of CYP enzyme production is not relevantly affecting CP levels. Instead, the observed increase in heme protein production appears to be compensated by a reduced breakdown of already existing heme, as indicated by the decrease in total bilirubin levels.

Overall, this study further supports CPI as a reliable OATP1B1 biomarker. Particularly, treatment with the CAR‐inducer efavirenz does not influence CPI plasma levels in this study, although CPs are byproducts of heme biosynthesis.

## Author Contributions

L.P., J.K., M.G., and H.E.M.S. wrote the manuscript. L.P., J.K., W.S., and H.E.M.S. designed the research. L.P., I.S., and J.K. performed the research. L.P., J.K., M.G., and H.E.M.S. analyzed the data.

## Funding

The authors have nothing to report.

## Conflicts of Interest

The authors declare no conflicts of interest.

## Supporting information


**Data S1:** cts70526‐sup‐0001‐supinfo.pdf.
