# Supplementary material for: Impact of Efavirenz on Heme Protein Induction and Biomarkers: Insights From In Vitro Experiments and a Clinical Study
Source: Clin Transl Sci. 2026 Mar 13;19(3):e70526. doi: 10.1111/cts.70526 (PMC13093287; doi:10.1111/cts.70526)
Supplement: Supplementary file 1 — Data S1: cts70526‐sup‐0001‐supinfo.pdf. [file CTS-19-e70526-s001.pdf]

Table S1

Table S1: Information on specific primers used for mRNA analysis by SYBR-Green based real-time PCR.

Gene name	Real-time PCR primer pair	Product size	aA Temp	aE Temp
**CYP2B6**	Forward: 5'-CAGCCACCAGAACCTCAACC-3' (300 nM)<br>Reverse: 5'-AAGGTCGGAAAATCTCTGAATCTCATA-3' (300 nM)	2400 bp	60°C (60 s)	
**CYP3A4**	Forward: 5'-ATCATTGCTGTCTCCAACCTTCAC-3' (300 nM)<br>Reverse: 5'-TGCTTCCCGCCTCAGATTTCTC-3' (300 nM)	368 bp	60°C (60 s)	
**ALAS1**	Forward: 5'-GAAACAGCCGAGTGCCAAAG-3' (300 nM)<br>Reverse: 5'-AGGGGTCAGATCTTTGCAGC-3' (300 nM)	81 bp	60°C (60 s)	
**18S rRNA**	Forward: 5'-GCAATTATTCCCCATGAACG-3' (300 nM)<br>Reverse: 5'-GGCCTCACTAAACCATCCAA-3‘ (50 nM)	123 bp	60°C (60 s)	

Figure S1

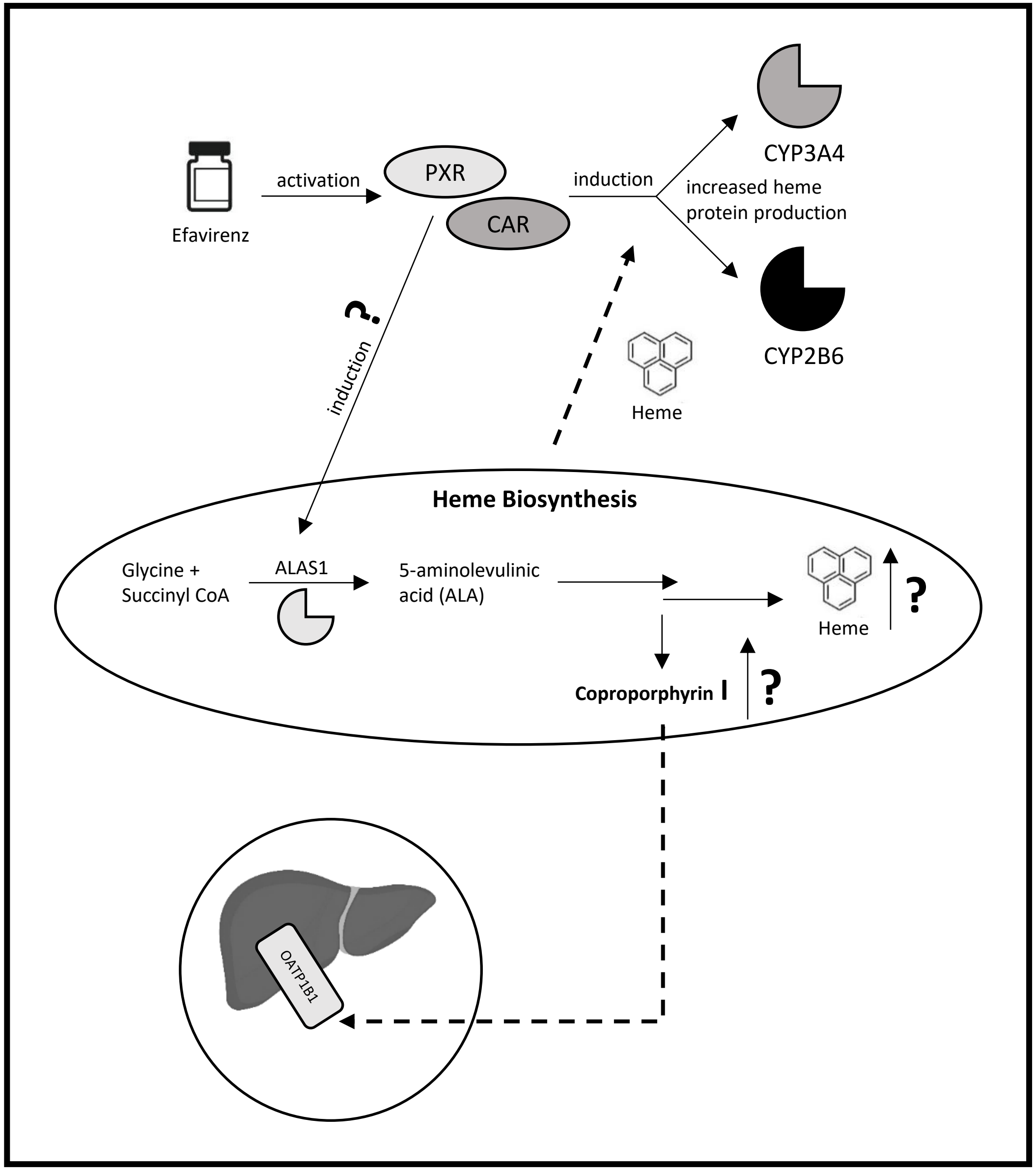

Figure S1: Systematic overview of key relationships within the study. PXR, Pregnane X receptor; CAR, Constitutive androstane receptor; ALAS1, 5-aminolevulinate synthase 1; OATP1B1, Organic Anion Transporting Polypeptide 1B1.

Figure S2

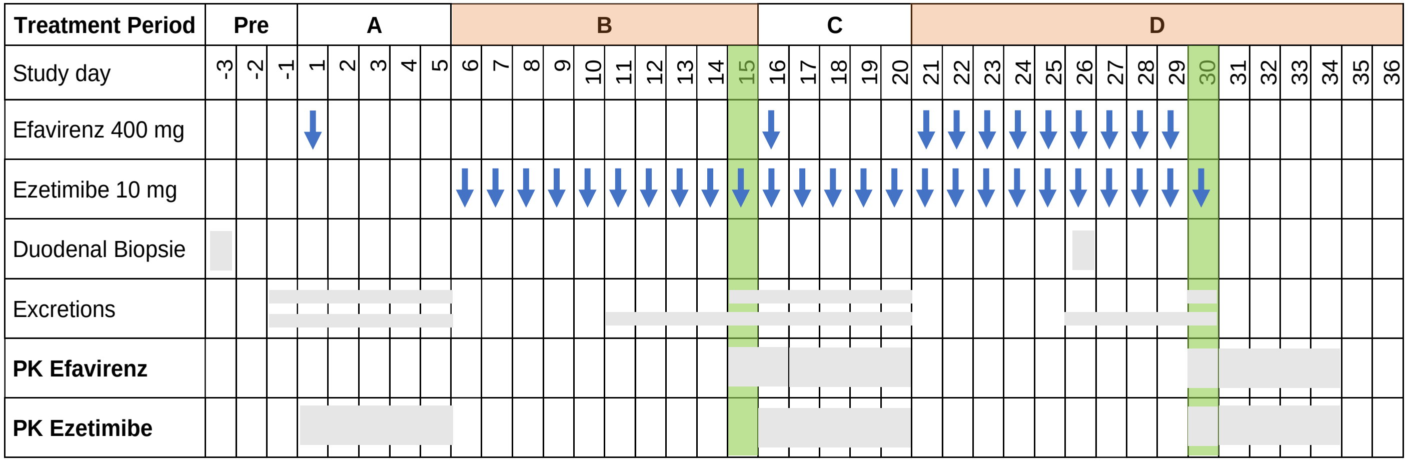

Figure S2: Gantt chart of the study design adapted from Oswald *et al.*, 2012. PK, pharmacokinetic. Arrows depict administration of the respective drug. Treatment periods from which samples were used for the analyses are marked in orange, associated study days are marked in green.

Figure S3

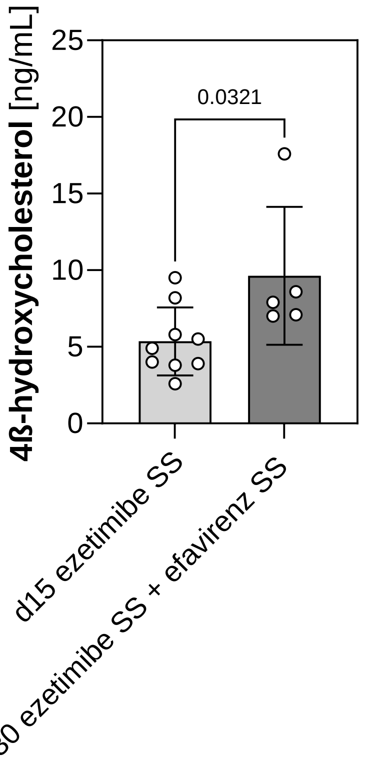

Figure S3: 4β-hydroxycholesterol plasma levels pre- and post-efavirenz induction. 4β-hydroxycholesterol plasma levels were determined in volunteers before and after oral treatment with efavirenz (400 mg once daily) for 9 days. Reported are 4β-hydroxycholesterol levels in the steady state of ezetimibe (d15 ezetimibe SS) and in the steady state of ezetimibe and efavirenz (d30 ezetimibe SS + efavirenz SS). Data are presented as individual values and mean ± SD. An unpaired *t*-test assuming equal variances was used to evaluate differences; p=0.0321.

Figure S4

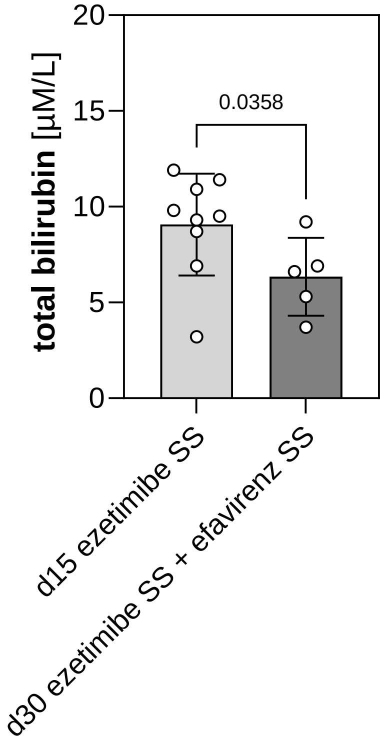

Figure S4: Total bilirubin plasma levels pre- and post-efavirenz induction. Total bilirubin plasma levels were determined in volunteers before and after oral treatment with efavirenz (400 mg once daily) for 9 days. Reported are total bilirubin levels in the steady state of ezetimibe (d15 ezetimibe SS) and in the steady state of ezetimibe and efavirenz (d30 ezetimibe SS + efavirenz SS). Data are presented as individual values and mean ± SD. An unpaired *t*-test assuming equal variances was used to evaluate differences; $p=0.0358$.
